# Male gender preference, female gender disadvantage as risk factors for psychological morbidity in Pakistani women of childbearing age - a life course perspective

**DOI:** 10.1186/1471-2458-11-745

**Published:** 2011-09-29

**Authors:** Farah Qadir, Murad M Khan, Girmay Medhin, Martin Prince

**Affiliations:** 1 Department of Behavioural Sciences, Fatima Jinnah Women University, Rawalpindi, 46000, Pakistan; 2 Department of Psychiatry, Aga Khan University, Stadium Road, Karachi 74800, Pakistan; 3 Aklilu Lemma Institute of Pathobiology, University of Addis Ababa, Addis Ababa, Ethiopia; 4 Institute of Psychiatry, King's College London, De Crespigny Park, London, SE5 8AF, UK

## Abstract

**Background:**

In Pakistan, preference for boys over girls is deeply culturally embedded. From birth, many women experience gendered disadvantages; less access to scarce resources, poorer health care, higher child mortality, limited education, less employment outside of the home and circumscribed autonomy. The prevalence of psychological morbidity is exceptionally high among women. We hypothesise that, among women of childbearing age, gender disadvantage is an independent risk factor for psychological morbidity

**Methods:**

A cross-sectional catchment area survey of 525 women aged 18 to 35 years living in Islamabad and Rawalpindi. The effect of gender disadvantage was assessed as a latent variable using structural equation modelling. Indicators were parental gender preference, low parental care, parental overprotection, limited education, early age at marriage, marital dissatisfaction and low autonomy. Psychological morbidity was assessed using the 20 item Self Reporting Questionnaire (SRQ).

**Results:**

Gender disadvantage was independently predictive of psychological morbidity. Among married women, socio-economic status did not predict psychological morbidity, and the effect of education was mediated through gender disadvantage rather than socioeconomic status (SES). The women's own preference for a male child was strongly predicted by their perceptions of having been disadvantaged by their gender in their families of origin.

**Conclusions:**

The high prevalence of psychological morbidity among women in Pakistan is concerning given recently reported strong associations with low birth weight and infant stunting. Social action, public policies and legislation are indicated to reduce culturally embedded preferences. Neglect of these fundamentals will entrench consequent inequities including gender bias in access to education, a key millennium development goal.

## Background

Women in Pakistan are particularly likely to suffer from depression and other common mental disorders. Prevalence in men is similar to that in other regions, but that in women is strikingly high; commonly more than half have clinically relevant symptoms [1-4]. Women in Pakistan are two to three times likelier than men to suffer from common mental disorders [5], compared with a typical female to male gender ratio of 1.5 to 2.0 elsewhere [6,7]. Socio-cultural rather than biological factors must be implicated. In this paper we focus upon the role of gender disadvantage, arising from the culturally determined predisposition to think about or behave differently towards women on the basis of their sex.

Male gender preference is deeply embedded in the culture of some countries [8]. Boys carry the family name, can continue the family trade, and are expected to provide for their parents in old age. Married women typically live with their in-laws, and are expected to provide care and support to their husband's parents in their old age. Married sons are therefore a virtual necessity in countries with no state pension or welfare support for frail older persons. Conversely daughters are lost to their family of origin. The starkest indicator of male preference is the one hundred million 'missing women' worldwide [9,10]. Slightly more boys than girls are born in every part of the world. Given equal care girls are hardier, so women predominate in most world regions; in Europe and North America there are 104 women for every 100 men in the population. However, in Pakistan (91 women for every 100 men), India (92/100), China (94/100), Bangladesh (94/100), and certain regions in North Africa (97/100) the opposite is the case. Female infanticide is an acknowledged but rare problem. More prosaically, girl babies are given less care, nourishment and access to family resources than their brothers. They therefore experience higher mortality.

In Pakistan medical care is sought for children more frequently than for women, but more for sons than daughters [11]. Critically ill male children were twice as likely as girls to be treated at hospital [12]. In Indian Punjab [13], girls were breastfed for a shorter time than boys, received less high prestige food, and received medical attention later. The neglect of girls extends into later childhood and adolescence. In Pakistan, only 25% of women, compared with 49% of men have completed primary education [14]. In urban Punjab, across all socio-economic strata female literacy is only around two-thirds that for men. For uneducated girls, 31% of parents 'did not agree' with the child attending school, compared with 7% of parents of uneducated boys [14]. One reason for undervaluation of daughters is the cost of marriage incurred by her family [15]. It seems plausible that unfavoured daughters will be married off heedlessly and relatively young by their families [16]. Early marriage limits educational opportunity, autonomy and financial independence. Women constitute only 28% of the Pakistani labour force [17]. Female mortality during peak child bearing years (20-29 years) is twice as high as that for men of the same age [18].

In summary, in Pakistan gender roles are exceptionally clearly defined. Son preference and daughter neglect prevails from birth [15]. Gender disadvantage has pervasive effects across the life course, much of it mediated through poor care and restricted opportunity. Pakistani women also seem to have exceptionally poor mental health. Previous studies have sought to quantify the impact of gender disadvantage by comparing outcomes between boys and girls, with the implicit assumption that girls were generally disadvantaged. We have limited our research to women (a population-based cross sectional survey of 525 women aged 18 to 35 living in four catchment areas in the twin Pakistani cities of Islamabad and Rawalpindi), assuming that the degree of gender disadvantage experienced varies among women and that this can be measured. We first used a path analysis to test our theory-driven model that gender disadvantage is an evolving life course phenomenon initiated by parental preference for a boy, with negative implications for parenting style, educational opportunities, marriage prospects, marital satisfaction and autonomy as an adult. We then sought

a) to quantify gender disadvantage as a latent variable with these multiple indicators, using structural equation modelling, and

b) to use structural equation modelling to test the hypothesis that there is an independent association between gender disadvantage and risk for psychological morbidity.

c) to explore whether the women's' experience of disadvantage because of their gender influenced their own preference for male over female children.

## Methods

### Study design, setting and participants

We conducted a population-based cross-sectional catchment area survey in Rawalpindi and Islamabad in northern Pakistan, purposively selecting one higher socioeconomic status urban catchment area (Westridge ward in Rawalpindi and Sector Division F-7 subsector 4 in Islamabad) and one lower socioeconomic status urban catchment area (Tenchbhata ward in Rawalpindi and Sector Division G-6 subsector 2 in Islamabad) from each of the twin cities. The catchment areas covered approximately one square kilometer. All female residents aged 20 to 35 years were eligible to be included. Enumeration of each catchment area was carried out systematically, street by street, starting at one border of the catchment area until the target of 125 eligible persons for each catchment area was recruited. In the low SES Rawalpindi area, following negotiation with community leaders and in the interest of good community relations, we completed door-knocking of the whole geographically defined target area resulting in a larger sample of 150 women. Participation was on the basis of informed signed consent, to be interviewed in private. The field research team consisted of FQ and four psychology Masters students. All interviewers were bilingual in English/Urdu and had previous field-work experience. After training in the structured interview methods, each interviewer completed 30 pilot interviews in a run-in phase with close field supervision, and co-rating of interviews until reliability was attained.

### Ethical Consideration

All the formal ethical procedures were followed while doing the study. The ethical approval was taken from the Institute of Psychiatry, King's College London and the South London and Maudsley (SLAM), National Health Service (NHS) Trust Ethics Committee (Research) in 2001

### Measures

Socio-demographic status: Age in years, current living arrangements (joint, partly joint, not joint), marital status (not married, engaged to be married/married/separated or divorced), and fertility (parity, interval between marriage and first child, fertility problems yes/no, number of abortions).

Psychiatric morbidity: The Self Rating Questionnaire (SRQ-20) it is a 20 item self-report scale based measure of psychiatric morbidity developed and validated cross-culturally as a psychiatric screening tool for primary health care settings in developing countries [19]. It has been translated into Urdu and used, and validated in four studies in Pakistan [4,20-22]. Case criteria for the study was set at ≥ 8 cutpoint on SRQ-20 [21]. When validated against the Present State Examination (PSE) as a second stage assessment of psychiatric morbidity, the sensitivity varied between 73%-83%, and specificity between 72%-85% [20]. When validated against the Psychiatric Assessment Schedule; the best threshold for women was 7/8 with a sensitivity of 78% and specificity of 81% [21].

Gender disadvantage:

1) Perceived gender preference of parents from birth. A parental preference for boys over girls, and a preferential allocation of care and family resources towards boys are the two most commonly cited elements of gender disadvantage in countries where the bias is culturally embedded. We asked

i) Did you feel that your parents would have preferred you to have been a boy? (Yes/no)

ii) Did you feel that your parents favoured your brothers or other male relatives over you? (Yes/no)

iii) Did you have the same access to health care and education as your brother or other male relatives? (Yes/no)

These questions were easily comprehended by Pakistani women and deemed appropriate to elicit experiences of disadvantage.

2) The Parental Bonding Instrument (PBI) [23] assesses the adequacy of a child's bonding with parents in early life in two dimensions; care and overprotection. Scale scores ascertained in adulthood reflect childhood experience [24], remain stable over long periods [25] and are relatively little coloured by current affective status [25,26]. We used the shorter 16 item version of the PBI [27], which we had previously validated in the same research setting [28]. We hypothesized that women disadvantaged by their gender would report lower levels of care and higher overprotection.

3) Level of education, collapsed into none, primary (up to 5 years), middle (up to 8 years), matriculation (up to 10 years), intermediate (up to 12 years), Bachelor's, Masters and Doctoral. We hypothesized that women disadvantaged by their gender would have attained a lower educational level.

4) For married women only

i) Age at marriage (in years)

ii) Marital satisfaction: The shorter 14 item Marital Satisfaction Scale [29] with a modified three point item response scale, as validated in an earlier pilot study in the same setting [30]. We hypothesized that women disadvantaged by their gender would have been married earlier and would report lower levels of marital satisfaction.

5) Autonomy. We included an ad hoc measure to assess the participants' degree of liberty and autonomy in adulthood in their parents' or husband's household. This was based on yes/no answers to the following four items (Cronbach's alpha, 0.76):

i) do your parents/husband restrict you going out with your friends?

ii) would your parents/husband allow you to work outside of home?

iii) would your parents/husband allow you to work in a male environment?

iv) if you like a man would you tell your parents? (for married women this was asked in the past tense)

We hypothesized that women disadvantaged by their gender would report lower levels of autonomy.

Socioeconomic status (SES): To ensure adequate variance in SES, sampling was stratified into two sets of catchment areas characterized by differing economic circumstances. For each participant, we also assessed current SES and SES of the family of origin as follows: household income, husband's income, father's income and a household assets index based on possession of the following utilities and items: television, video recorder, computer, air conditioning, car, domestic help, visits abroad, home owner, number of bedrooms, number of bathrooms. Cronbach's alpha was 0.89.

Life Events: The List of Threatening Events [31] identifies 12 event categories most likely to be rated as providing significant contextual threat. We ascertained events occurring in the last one year.

Social Support/Social Network: Close Persons Questionnaire (CPQ) [32] is a structured questionnaire assessing several dimensions of social support, including emotional/confiding, practical and negative aspects of support from up to four identified sources. The Close Persons Questionnaire has been used on Asian population including Pakistanis living in the United Kingdom [33] however it has not been previously used in Pakistan.

Woman's gender preference for her own child: assessed using the question 'How important is it for you that your first child should be a son?' with the responses 'not so important', 'important' and 'very important'.

### Analysis

We report the principal characteristics of the sample by catchment area. Next we conducted a pathway analysis, to test the model that gender disadvantage is a life course phenomenon with pathways from parental preference for a boy to low care and high overprotection, to low educational level, to early age at marriage and low marital satisfaction to low levels of autonomy in adulthood. Each component is represented by an observed variable. The full model could be run on married women only. A model omitting age at marriage and marital satisfaction was run in a secondary analysis on the whole sample. Next we developed a structural equation model to test the hypothesis that gender disadvantage is independently associated with psychological morbidity. The measurement model initially consisted of five measurement sub-models for the following unobserved variables (latent constructs)

1) psychological morbidity, for which the indicators were the 20 SRQ items

2) negative marital satisfaction (the seven negatively orientated marital satisfaction items)

3) positive marital satisfaction (the seven positively orientated marital satisfaction items)

4) socio-economic status (high versus low SES catchment area, household assets index, husband's income, father's income, household income and level of education)

5) gender disadvantage (the three gender disadvantage perception questions, PBI care, PBI overprotection, level of education, age at marriage and autonomy).

Level of education was therefore an indicator for socioeconomic status and gender disadvantage. Positive marital satisfaction, negative marital satisfaction, socioeconomic status and gender disadvantage were all assumed to be correlated with each other. We tested for pathways from socioeconomic status and gender disadvantage to psychological morbidity. We also tested for direct pathways from each of the gender disadvantage indicators to psychological morbidity (i.e. effects of the observed variable not mediated through the latent construct of gender disadvantage), and for direct effects of age, number of children, interval between marriage and first child, fertility problems, number of abortions, number of life events and emotional/confiding social support. Non-significant pathways and variables were removed from the model. Again, the full model could only be run on married women. A partial model, excluding age at marriage and marital satisfaction, was run on the whole sample as a secondary sensitivity analysis. Pathway associations for the prediction of psychological morbidity are expressed as crude and standardized regression weights. Correlations are reported for associations between unobserved variables. A squared multiple correlation indicates the proportion of the variance in the latent construct psychiatric morbidity accounted for by the variables in structural model. Model fit was assessed using the Tucker-Lewis index (TLI), Root Mean Square Error of Approximation (RMSEA) and Akaike's Information Criterion (AIC). Akaike's Information Criterion (AIC) [34] adjusts the model chi-square to penalize for model complexity. The lower the AIC value, the better the fit of the model [35]. The Tucker-Lewis index (TLI)[36] indicates the proportion of co-variation among indicators explained by the model relative to a null model of independence, and is independent of sample size. Values near 1.0 indicate good fit; those greater than 0.90 are considered satisfactory [37,38]. The Root Mean Square Error of Approximation (RMSEA) assesses badness of fit per degree of freedom in the model and is zero if the model fits perfectly; RMSEA values of less than 0.05 indicate close fit and 0.05 to 0.08 reasonable fit of a model [39].

Finally, across the whole sample, we used ordinal regression to identify independent associations between socio-demographic, socioeconomic and gender disadvantage variables and the extent of the woman's preference that her first child should be a boy.

## Results

Four hundred and eighty six households yielded a total of 551 eligible women; 525 interviews were completed with a response rate of 95.2%. All of the interviews were carried out in private although very occasionally female relatives would sit in for part of the interview. Twenty-one women (3.8%) declined to participate, thirteen because they were refused permission by relatives to participate, eight chose not to do so for themselves. Five women could not be contacted for interview. Three hundred and four women were married (58.0%). The overall prevalence of likely psychological morbidity (an SRQ 20 score of 8 or over) was 55.4%, varying from 26.4% (high SES Rawalpindi) to 82.7% (low SES Islamabad). After adjusting for city, women living in the low SES areas were more likely to have psychological morbidity (Table 1). They were also more likely to be married and less likely to work outside of the home. Among the hypothesized indicators of gender disadvantage, those living in low SES areas were more likely to perceive gender disadvantage, had lower care scores, higher overprotection scores, less education, earlier age at marriage, lower marital satisfaction, and lower degrees of autonomy. They were more likely to report life events, and received less emotional support. For married women, those living in low SES areas had more children and were less likely to lack a son.

**Table 1:** Sample characteristics in the four catchment areas, with the effects of Socio Economic Status (SES), adjusting for city

*Catchment area*	*Low SES* *Islamabad*	*High SES Islamabad*	*Low SES Rawalpindi*	*High SES Rawalpindi*	
	**N = 150**	**N = 125**	**N = 125**	**N = 125**	**Effect of SES** **(Low vs. High - Mantel-Haenszel odds ratios with 95% CI)**

**Sociodemographic circumstances**					
Age in years, mean (SD)	27.1 (5.2)	25.2 (4.8)	26.5 (4.8)	26.8 (4.6)	
Married	102 (68.0%)	54 (43.2%)	74 (59.2%)	74 (59.2%)	1.7 (1.3-2.5)
Nuclear (vs. joint) household	112 (74.7%)	66 (52.8%)	57 (45.6%)	57 (45.6%)	1.6 (1.1-2.3)
Employed outside of the home	18 (12.0%)	30 (24.0%)	23 (18.4%)	33 (26.4%)	0.5 (0.3-0.8)
**Psychological morbidity**					
SRQ score > = 8	124 (82.7%)	56 (44.8%)	78 (62.4%)	33 (26.4%)	5.2 (3.5-7.6)
SRQ score, mean (SD)	11.4 (4.1)	7.6 (3.6)	9.6 (5.0)	5.3 (3.5)	F = 125.7, P < 0.001
**Gender disadvantage**					
Parents would have preferred a boy	69 (46.0%)	34 (27.2%)	87 (69.9%)	27 (21.6%)	4.0 (2.8-5.9)
Parents favoured male relatives	18 (12.0%)	13 (10.4%)	67 (53.6%)	12 (9.6%)	4.2 (2.6-6.7)
Male relatives had more access to health and education	17 (11.3%)	4 (3.2%)	10 (8.0%)	4 (3.3%)	3.2 (1.4-7.3)
Parental Bonding Interview Care, mean (SD)	11.2 (2.4)	14.6 (3.9)	11.7 (2.4)	16.6 (3.4)	F = 226.2, P < 0.001
Parental Bonding Interview Overprotection, mean (SD)	22.1 (3.6)	15.2 (3.3)	22.1 (3.1)	16.0 (3.8)	F = 450.3, P < 0.001
Education (ten years or less)	121 (80.6%)	7 (5.6%)	67 (53.6%)	2 (1.6%)	70.6 (32.7-152.3)
Age at marriage in years, mean (SD)	18.7 (1.5)	23.6 (3.1)	19.3 (1.9)	22.8 (1.8)	F = 300.6, P < 0.001
Marital satisfaction, mean (SD)	-4.3 (7.0)	-0.7 (8.5)	-4.2 (9.2)	3.1 (9.3)	F = 30.5, P < 0.001
Autonomy, mean (SD)	1.0 (0.9)	2.8 (1.2)	1.4 (1.1)	2.4 (1.2)	F = 73.8, P < 0.001
**Socioeconomic status**					
Household income, mean (SD)	7300 (3403)	66592 (47689)	9918 (3850)	47705 (31891)	F = 140.4, P < 0.001
Household wealth index, mean (SD)	1.7 (1.5)	7.5 (2.7)	2.9 (1.8)	7.8 (2.1)	F = 310.4, P < 0.001
**Other potential confounders**					
Life events (one or more)	107 (71.3%)	79 (63.2%)	93 (74.4%)	77 (61.6%)	1.6 (1.1-2.3)
Confiding/emotional support, mean (SD)	22.1 (3.8)	25.1 (4.4)	23.6 (3.7)	25.9 (4.0)	F = 56.7, P < 0.001
Number of children, mean (SD) (married women only)	3.1 (1.7)	1.7 (1.4)	2.4 (1.7)	2.0 (1.4)	F = 11.8, P < 0.001
No son (married women only)	20 (19.6%)	27 (50.0%)	25 (33.8%)	28 (37.8%)	0.5 (0.3-0.8)

**Figure 1:**
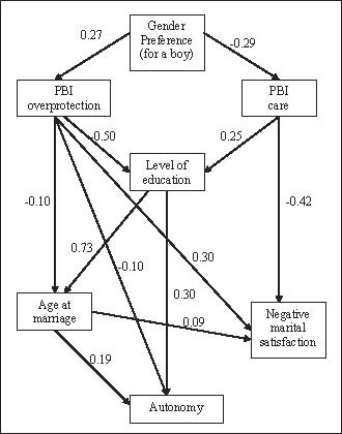
**Pathway diagram for associations between hypothesized markers of gender disadvantage, with standardized regression weights**.

**Figure 2:**
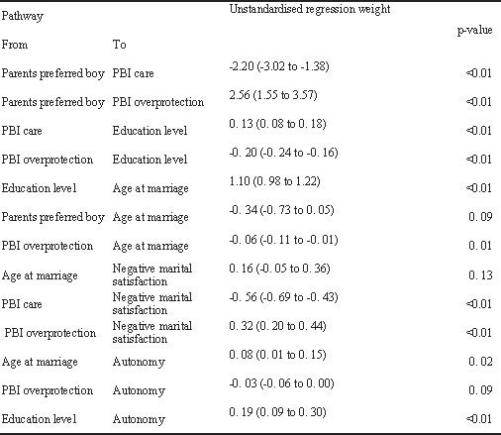
**Parameters for pathway model (Figure 1)**.

The pathway analysis, testing the hypothesised model for gender disadvantage as a life course phenomenon, indicated a complex pattern of strong and statistically significant associations (Figure 1 and 2). Each variable was associated with the subsequent variable in the causal chain and effects on more distal variables were mediated indirectly through the intervening variables. Thus perceived parental preference for a boy was associated with PBI overprotection and low PBI care, which were in turn associated with low educational level. The direct effect on education of parental preference for a boy was negligible. Low PBI care predicted marital dissatisfaction whereas overprotection directly predicted both early age at marriage and marital dissatisfaction. There was a very strong direct effect of (low) education on (early) age at marriage, and a less substantial effect on autonomy. The pathway analysis for the whole sample necessarily omitted age at marriage and marital satisfaction. Other pathways were consistent with those reported for the subset of married women (data not shown but available from the authors on request).

**Figure 3:**
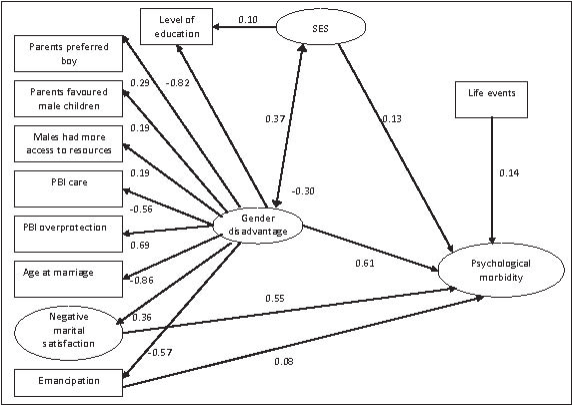
**Structural Equation Model**.

**Figure 4:**
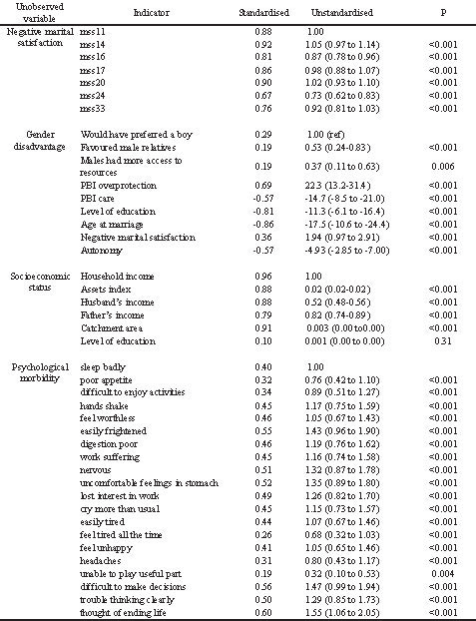
**Measurement model for structural equation model (Figure 3)**.

Our structural model for married women (Figure 3, Figure 4 and Table 2), with the latent variable 'psychological morbidity' as the outcome revealed a strong independent effect of gender disadvantage (standardized beta weight 0.61, p < 0.001) upon psychological morbidity. There was also a strong direct effect of negative marital satisfaction (0.55, p < 0.001) and a modest effect of life events (0.14, p = 0.003). Positive marital satisfaction (a latent variable), age, social support and all of the fertility variables dropped out of the model. Gender disadvantage was highly correlated with SES (-0.87). However, the effect of education (specified as an indicator of SES as well as gender disadvantage) on psychological morbidity was mediated entirely through gender disadvantage. The effect of SES was negligible (beta weight 0.13) and statistically insignificant (p = 0.24). Other than marital dissatisfaction, none of the indicators of gender disadvantage (with the possible exception of autonomy 0.08, p = 0.12) had direct effects upon psychological morbidity, not mediated through gender disadvantage. Overall model fit was good (AIC 1748.1, TLI = 0.87, RMSEA = 0.05). For the whole sample, including unmarried women we tested the same model excluding the marital satisfaction latent variable and age at marriage observed variable. The model fit was again adequate (AIC = 1548.6, TLI = 0.85, RMSEA = 0.06). The most notable difference was that SES was now independently associated with psychological morbidity (standardized beta weight 0.61, p < 0.001) together with gender disadvantage (1.46, p < 0.001), social support (-0.15, p < 0.001), life events (0.20, p < 0.001) and a direct effect of autonomy (0.20, p = 0.002). Parameter estimates for the sub-sample of single women were very similar to those for the sample as a whole (data available on request) suggesting that any differences in the parameter estimates between the married women and the whole sample arose from the necessary differences in the model specifications rather than differences in the pattern of associations between married and single women. The substantial independent effect of gender disadvantage on psychological morbidity is apparent in both marital status strata as well as in the whole sample.

**Table 2:** Coefficients from structural equation model

Pathway		Married women only (n = 304)			All women (n = 525)		
From	to	Standardised	Unstandardised	p-value	Standardised	Unstandardised	p-value
Gender disadvantage	Psychological morbidity	0.61	0.81 (0.30 to 1.32)	0.002	1.46	1.15 (0.67 to 1.63)	< 0.001
Socioeconomic status	Psychological morbidity	0.13	0.00 (0.00 to 0.00)	0.24	0.61	0.00 (0.00 to 0.00)	< 0.001
Negative marital satisfaction	Psychological morbidity	0.55	0.13 (0.09 to 0.17)	< 0.001	n/a	n/a	n/a
Autonomy	Psychological morbidity	0.08	0.01 (-0.01 to 0.03)	0.12	0.20	0.02 (0.01 to 0.04)	0.002
Life events	Psychological morbidity	0.14	0.02 (0.01 to 0.03)	0.003	0.20	0.02 (0.01 to 0.03)	< 0.001
Social support	Psychological morbidity	-	-	-	-0.15	-0.01 (-0.01 to 0.00)	< 0.001
Correlation between		Correlation	Covariance (95% CI)	p-value	Correlation	Covariance (95% CI)	p-value
Gender disadvantage	Socioeconomic status	-0.87	-18.7 (-10.8 to -26.7)	< 0.001	-0.86	-24.1 (-18.1 to -30.1)	P < 0.001

In the ordinal regression predicting the woman's preference for a male child, 39.8% of women said that it was 'not so important' that their first child was a boy, 44.0% said it was 'important', and for 16.2% it was 'very important'. Neither age, education, marital status, household income, nor SES area was associated with stronger preference for a male child, and these variables dropped out of the model. The final parsimonious predictive model included only indicators linked to the mother's own experience of gender disadvantage; her perception that her parents would have preferred for her to have been a boy (odds ratio 1.67, 95% confidence intervals 1.15 to 2.38), her perception of lack of parental care (OR 0.86, 95% CI 0.81 to 0.91) and parental overprotection (OR 1.07, 95% CI 1.03 to 1.12). The model provided a good fit; chi squared 530, 515 df, p = 0.31; Nagelkerke pseudo R^2 ^= 0.21.

## Discussion

Women's' health is inextricably linked to their status in society, benefiting from equality and suffering from discrimination [40]. Gender influences many of the determinants of mental health, including socioeconomic position, access to resources, social roles, rank and status. Gender differences in mental health outcomes diminish after controlling for social differences between men and women [6,41]. There is therefore good reason to suppose that the effect of gender is, at least in part, mediated through disadvantages that women experience because of their sex. We set out to estimate the effect of gender disadvantage as an individual attribute upon psychological morbidity among women of child-bearing age in urban Pakistan.

This was a population-based study with whole catchment area samples, and a very low proportion of non-responders. We have shown that it is possible to interview women privately in this male authoritarian culture, a necessary precondition for unbiased study of indicators of gender disadvantage. The main outcome, the SRQ-20 had been validated, and used extensively, in previous population-based research in Pakistan. Our measures of parental bonding and marital satisfaction had been validated in our earlier pilot study [28,30]. The main weakness was the cross-sectional design, with exposures assessed synchronously with outcome. Markers of gender disadvantage across the life course were ascertained through retrospective recall and information bias cannot be excluded. The path analysis and, to a lesser extent, the structural equation models assumed, implicitly, a temporal ordering of causal relationships; the overall model fit for the structural equation models was adequate to good in terms of the RMSEA, but slightly sub-optimal (< 0.90) in terms of the TLI.

Our pathway analyses supported gender disadvantage as an evolving life course phenomenon with strong effects of parental preference for males on care and overprotection; of care and overprotection on limited education and negative marital satisfaction; of limited education and overprotection on early age at marriage; and of limited education and early age at marriage on autonomy. These variables constituted the indicators for a latent trait of gender disadvantage, used in the subsequent structural equation model to predict psychological morbidity. Research evidence links each of these variables with poor mental health outcomes, including some studies from Pakistan. Adverse childhood experiences are associated with an increased risk of adult psychiatric disorder [42-44]. In Pakistan, parenting may be particularly salient to women's mental health, given the prevailing male gender preference. A daughter who is little valued in her family of origin may internalize this into her cognitive schema, carrying this disadvantage throughout her life and making her vulnerable to continuing neglect or abuse. Gender disadvantage may be transmitted to the next generation of daughters in a self-perpetuating cycle; in our study the mother's perception that her parents would have preferred for her to have been a boy, her perception of low parental care and high parental overprotection were each independently and strongly associated with her own preference for her first child to be a boy. Some caution is required in transferring western concepts of 'good' and 'bad' parenting to other settings; there is more parental control, and control is more tolerated in Asian cultures, including Pakistan [45]. However, we had previously validated the PBI for use in this population, finding, despite theoretical concerns, that overprotection was strongly correlated with lack of care and that each was robustly associated with psychological morbidity [28]. Our study is the first in Pakistan addressing the association between parenting and adult psychological morbidity, and the first anywhere to link lack of parental care and overprotection, in women, to the experience of gender disadvantage.

Several cross-sectional epidemiological studies in Pakistan have reported an association between level of education and psychological morbidity [46,47]. A striking finding from the current study is that, in the structural equation model, education loads more heavily on the gender disadvantage latent variable than the SES latent variable, and that the effect of education on psychological morbidity is mediated entirely through gender disadvantage. Education, when considered as a risk factor for psychological morbidity in low income countries is often construed mainly as a marker of socio-economic disadvantage [48]. However, education empowers women and counters culturally-embedded disadvantage; better educated women choose to marry later, are more likely to be involved in family planning [49], earn more [50] and exercise greater control over household resources. Female educational disadvantage is also self-perpetuating; in Pakistan the gender bias in access to education is most pronounced for children of women of lower status [51].

As far as we are aware, no previous studies have assessed subjective marital satisfaction among Pakistani women. Several studies suggest that this is an apt area for investigation, with a high prevalence of intimate partner violence [52,53], and associations between psychiatric morbidity and intimate partner violence [53], absence of confiding relationship with husband [54], and arguments with husband and in-laws [55]. Married women predominate among those attempting suicide [16], with the most frequently reported reasons being conflict with husband or in-laws. The construct validity of the Marital Satisfaction Scale was demonstrated in a pilot study prior to the current survey [30]. As in the main survey we found a wide distribution of scores with a significant proportion of women prepared, when interviewed privately, to express dissatisfaction.

While the tentacular influence of gender disadvantage in Pakistan may plausibly explain the marked excess of psychological morbidity among women, this postulate, the starting point for our research, is not directly testable using our study design. Our findings are consistent with those from a recent large cross-sectional survey in Goa, South India with strong associations between common mental disorder and several indicators of gender disadvantage including early age at marriage, intimate partner violence and abuse, and lack of decision-making autonomy [56]. We have provided empirical evidence to support a model [57] for gender disadvantage starting when a girl child is born to parents who would have preferred a boy, with profound, cumulative consequences across the life course. While gender disadvantage and poverty are strongly correlated, our findings suggest that the former is more directly relevant to the epidemic of mental ill health in Pakistani women. In this context, the strong independent associations recently reported from Pakistan and India between maternal depression and low birth weight [58,59], infant stunting [59,60] and impaired development [60], constitute a powerful argument for a coordinated public health response.

India has taken some steps to address the problem. The Dowry Law, enacted in 1964 outlawed this practice, which nevertheless remains widespread. The increasing availability of pre-natal determination of gender by ultrasound enables the selective abortion of female foetuses, a procedure thought to account for up to half a million 'missing' girl babies a year in India [61]. India again has strict laws - The Prenatal Diagnostic Technique (Regulation and Prevention of Misuse) Act of 1994. Doctors and parents who offend are subject to heavy fines and up to five months imprisonment. However, in practice the technique remains available to those who seek it. While legislation has its place there is a clear role for public policy and social action to counter the culturally embedded and self-propagating preference for boys over girls. Without a concerted, sustained attempt to address these fundamentals, the consequent gendered inequities in infant and child mortality, and access to education, the overcoming of which are key Millennium Development Goals, seem set to remain [62]. National action plans might also usefully consider evidence-based community [63,64], group [65,66] and individual interventions [67,68] targeting empowerment of women and mobilization of peer and community support. The former has been shown to be effective in reducing neonatal mortality rates by up to 30% [63,64] with some potential benefits for maternal depression [64], and the latter to have dramatically beneficial effects upon women's mental health [68].

## Conclusion

For women in Pakistan, gender disadvantage may originate from a parental preference for boys over girls, and then accumulate over the early lifecourse with important implications for future life opportunities, autonomy and adult mental health. In addressing issues concerning women and girl children, governments of countries where male gender preference is prominent should promote the mainstreaming of gender perspectives that emphasize equity into all policies and programs. In so doing, it may be helpful to highlight that the disadvantage that women experience because of their gender may be an important contributor to their high risk of psychological morbidity, and that this in turn may have important adverse consequences for child health and development.

## Competing interests

The authors declare that they have no competing interests.

## Authors' contributions

FQ and MP designed the study, analysed the data and jointly wrote the first draft of the manuscript. FQ led the field work in Pakistan. MK and MP supervised FQ's PhD studies, from which this manuscript originated. GM contributed substantially to the analysis, write-up and interpretation of the results. All authors have read and approved the final version of the MS for submission for publication

## Pre-publication history

The pre-publication history for this paper can be accessed here:

http://www.biomedcentral.com/1471-2458/11/745/prepub
